# The Therapy of Diarrhœa

**Published:** 1874-12

**Authors:** F. A. Hartsen

**Affiliations:** Marseilles


### THE THERAPY OF DIARRHŒA.

BY F. A. HARTSEN, (MARSEILLES.)

*From Virchow's Archives, August 17th,* 1874.

Preconceived opinions play as important roles in the treatment of diarrhœa as they do in that of albuminuria. We do not hesitate to express the conviction that half the sufferers from chronic diarrhœa die rather from the remedies employed in the treatment than from the disease. Especially is this true of tuberculous patients. The diarrhœas of phthisical cases are as a rule, at least in the beginning, of a simple catarrhal nature. In such cases where digestion—the last support of the patient—is now only two often disturbed by astringents and the like, a skillful treatment might bring about some improvement of the intestinal tuberculosis, and if they could not be entirely prevented, the formation of ulcers could at least be retarded. In the lazaret at Utrecht we have seen bodies of tuberculous patients opened, the inner surface of whose intestines was colored perfectly black by the long continued and unintermitting use of nitrate of silver. A perfect silver mine!

We cannot be too much upon our guard against the abuse of false inferences, both with reference to our especial subject, and to therapy in general. In connection with so complicated and mutable an object as our organism these can, *a priori*, but seldom find application.

If the unqualified *similia similibus* of consistent homeopathists is arbitrary, the unlimited *contraria cantrariis* of many allopaths is no less arbitrary and one-sided. Astringents (preparations of iron) for example, are a current remedy for diarrhœa. Wherefore? A healthy man, after taking large doses of them (muriate, tannate, etc.,) becomes constipated. Ergo, we argue, astringents are constipating agents, and consequently indicated in diarrhœa. But, alas, nature concerns herself but little about our philosophising. It is not necessary to repeat the experiment of giving large doses of iron to a man, suffering from a severe diarrhœa, for it has already been too often tried. Instead of being improved, the diarrhœa was always aggravated.

The operation of the preparations of iron has been observed by many physicians; but that of the vegetable astringents, as tannic acid, rhatany, etc., has not been at all or only insufficiently noticed. Even the simple bitters, as colombo and gentian, require great caution.

So firmly convinced are many physicians of the efficacy of astringents that they think, when these do not avail, the dose should be doubled. And if the diarrhœa does not then give way, the nature of the of the patient is made responsible for it. Astringents should help, it is logical, and it is so stated in the books*.

The French physicians originated the prepossession in favor of astringents by so highly recommending the use of Bordeaux-wine in diarrhœa. This is an extremely pernicious custom, the more so, as the name Bordeaux-wine is, as a rule, only a label for a problematical brewing, among the ingredients of which are not only tannin, but other organic acids.

Indeed, in case of an otherwise healthy man who has a common attack of diarrhœa we may give, so to speak, anything we choose.

*Niemeyer somewhere expresses the opinion that the utility claimed for the acetate of lead in the diarrhœa of tuberculosis depends in great part on the opium given with it. We think he is perfectly correct, and the same may be said of other astringents.

He will recover of himself, and that too, in spite of many injurious influences. Medicine is, however, required in the treatment of severe cases, and these demand the greatest caution.

In our opinion, in suspicious diarrhœas, the following classes of influences are contraindicated:

Everything which irritates the intestinal mucous membrane, directly or indirectly (through nervous influence.)

To these belong the astringents; acetate of lead, nitrate of silver and tannic acid. We think, perhaps, that some of the most common articles of food are rich in tannin, as tea, coffee, chocolate, cocoa, (even deprived of its oil); we should use these only in very small quantities.

The mineral acids belong also to the astringents. In the treatment of diarrhœa, sulphuric and hydrochloric acids require great caution. Preparations of calcium also act as astringents. Perhaps the use of lime-water should be limited to such cases as have an excess of acid to be neutralized.

Further, the organic acids are irritating to the intestinal mucous membrane.

We recommend entire abstinence from fruits and vegetables; in short, from all things containing organic acid. The acid of vinegar is pernicious even in small quantities. Attention should be paid to this fact in the preparation of food. Lemon juice is less injurious, but it too should be avoided. Wine must be rejected. We have seen nothing but harm result from the use of every variety of wine, even the much lauded Port and Malaga. Warm water, with a little rum or brandy, makes a good drink, provided the rum and brandy do not contain much acid.

Carbonic acid may, in a certain measure, be looked upon as a bridge between the organic and inorganic acids. This is decidedly irritant to the gastric and intestinal mucous membranes. We have frequently seen simple carbonic acid water increase diarrhœa.

Closely allied to the astringents are the simply "bitter tonics," as quinine, gentian, colombo, nux vomica. These agents, too, causing constipation in healthy individuals, may in irritable intestines provoke diarrhœa. They are also contraindicated in diarrhœa.

Should it be impossible to avoid the administration of astringents (iron, tannin, etc.,) or bitters (quiniæ sulph.), they should at least be given combined with opium.

Sulphate quinia is usually given in solution. The customary solvent, sulphuric acid, should preferably be replaced by muriatic acid, as the latter is less injurious to the stomach. Beer is very bad, not only on account of the carbonic acid, but also in several other respects (fermentation, acids, bitter materials, etc.)

We consider even salts and alkalies as capable of danger as irritants. We cannot concede that the purgative effects of the remedial salts depend simply on their physical influence (increased indosmosis.) We have observed that these not only increase the fluidity of the fæces, but also change their condition in other ways (their odor for instance.)

Bicarbonate of soda should only be prescribed in small doses. Common salt has the effect of continuing a diarrhœa; therefore the food should be prepared with as little salt as possible. Oysters, hams, herring, mineral waters, and the like, should be avoided by persons predisposed to diarrhœa—a precaution too often neglected by consumptives who have been sent to Sodom.

We caution against expectorants, as salammoniac, golden sulphur, iodide of potassium, etc.

We must not forget that meat broths usually contain a good deal of salt, only it is not noticed because the salt is distributed in such a large quantity of water. Liebig's extract of meat is decidedly aperient, and in many cases injurious to the stomach.

Further, the aromatic and empyreumatic remedies are irritating; in grave diarrhœa we should beware of using heating medicines and carminatives, as peppermint, ginger, aniseed, Eaundes Carmes, etc. Foods much spiced with pepper are injurious. We should use only sufficient spices to promote digestion.

The burnt products of tobacco are pernicious. Patients with diarrhœa should not smoke, or if they do, should not swallow their saliva, but spit it out, and should rinse out the mouth frequently.

Fats are irritating either directly or indirectly (as by the acid products of decomposition which they supply during digestion.) Moreover, many fats contain acrid constituents, as does that of olive oil. We have frequently met with a very acrid resin in unripe olives.

What has been said of the fats applies also to sugar. Sweets (syrups, etc.,) have at least as favorable influence on the diarrhœa.

Extremes of cold and heat may likewise be reckoned among the irritating influences. We never recommend cold food, and least of all, never cold drinks to those sick with chronic diarrhœa. It is always easy to add a little warm water to the water or milk, which we are about to drink. If nothing but cold drink can be obtained, it should be allowed to remain in the mouth until somewhat warmed.

These all seem trifles, and many of them being distasteful, it is doubtful whether they can be exactly carried out. Nevertheless, it is the duty of every sick person, as his life is of value to society somewhere.

Cold is indirectly injurious when it affects certain parts of the skin. The patient should keep his feet warm, and should always wear very warm under-clothing on the abdomen.

The outhouses deserve particular attention in a hygienic point of view. Scarcely anything is more pernicious in a diarrhœa than a draught of air against the lower portion of the trunk. The patient who notices the least draught in the opening of the privy should endeavor to help himself in some other way. In Germany, unfortunately, there is still much room for improvement in the arrangements of these outhouses.

It is scarcely necessary to remark that firm, hard masses irritate the intestinal mucous membrane. From this, however, follows the important precaution to carefully masticate all food, and if necessary to limit one's self to fluid, or half fluid nourishment; abstinence from bread with bran, chocolate, peas, etc.

Atmospheric and mental influences may cause variations in the activity of the intestines in an indirect manner (through the nervous system.) Constipation is produced in some few by a moist, and in others by a dry atmosphere. Mountain air has the reputation of being constipating, and is therefore to be recommended in certain forms of chronic diarrhœa (Lombard, *le climat des montagnes*, Geneve, 1874.)

Residence in the South is to be recommended. Those who are benefitted by a moist temperature should go to Pau; and those by pleasant dry weather should spend the winter on the coasts of the Mediterranean, avoiding, however, the immediate neighborhood of the water.

All catarrhal influences should be shunned by persons afflicted with diarrhœa. They must not go out at night, nor in bad weather. We must not consider the influence of the air to be indifferent because it does not come into direct contact with the intestines. Great heat is to be avoided.

Concerning the effects of moral influence, we know that severe mental shocks, especially anxiety, easily increase the morbid intestinal activity. So, too, does mental exertion.

A second class of influences which keep up, and may even produce diarrhœa, are those which may injure digestion. Their mode of operation can always be referred to improper food, or its improper preparation

A weak digestion is easily disordered by anything not easily digestible, and especially by those to which the normal organism is unaccustomed, as spices and medicaments. In patients with weak digestion, remedies should only be prescribed in solution, and that, too, a very weak solution. Concentrated solutions irritate the mucous membranes, and solid agents do so to a still greater extent. Before we enter upon the medicinal treatment of a long continued diarrhœa, there should be, in tolerably hearty persons, entire absence of food, and drink as long as it can be endured. If this cannot be done, they may be limited to the most easily digestible lean meats and farinaceous foods. Of the farinaceous substances those containing the most mucilage, as rice and barley, are to be preferred. Good arrowroot is to be recommended. No more should, however, be eaten of these than is necessary, as in digestion they are changed into sugar or lactic acid, and may, under some circumstances, increase the diarrhœa.

Milk diet is frequently recommended. Those doing so forget that: First, milk contains much water and affords insufficient nourishment; second, milk, especially the undiluted, is rich in fat; third, in diarrhœa milk may easily pass into the intestine before the stomach has sufficiently affected it; there (in the intestine) it undergoes an acid fermentation; fourth, milk must not be drank cold; fifth, milk demands great precautions relative to its freshness, origin, etc.

Boiled milk is always to be preferred to the unboiled, as it does not so readily undergo fermentation.

Combining in short the above observations, we have the following indicated:

Diarrhœa of whatever kind or produced by whatever causes depends, as a rule, upon an increased irritability of the intestinal canal. The augmentation of this irritation is to be avoided as much as possible. Instead of immediately administering the metallic preparations (as the salts of lead, zinc and bismuth*) and other astringents, we should first exhaust all the remedies which are appeasing. Warm applications (of the requisite breadth) to the abdomen can scarcely be sufficiently extolled. Of the remedies, opium should be the first considered, in connection with which it must be remembered that in large doses digestion is disturbed and the diarrhœa thus increased.

If the diarrhœa be so severe that the opium escapes from the organism without being absorbed, the hypodermic injection of morphia will be indicated. Clysters are liable to the objection that they irritate the large intestine; first by the instrument, secondly by the volume of injection. They should only be resorted to in cases of emergency.

We have already said sufficient concerning the diet.

Ipecac may be of advantage in case opium does not suffice.

Should all this be found insufficient, bismuth (subnitrate or oxide) may be tried; although an irritant, it may have some influence on the duration of the malady.

* The influence of bismuth is considered to be purely mechanical, acting as a simple coating which protects the mucous membrane from the contact of the food. This material is not, however, so simple and harmless, By the use of bismuth the excrement becomes black. The nitrate of bismuth is decomposed by the sulphur in the intestines. What, however, has become of the nitric acid? We consider nitrate of bismuth as an irritant.

Tonics and astringents are now to be considered in atonic diarrhœa (provided such exists). Where, however, there are the slightest irritations, as by cancer, tuberculosis, etc., they are absolutely inadmissible. The supposition that we can coat over intestinal ulcers by the administration of astringents we consider a false hypothesis. If the astringents were able to form a crust, the healthy portion of the intestine would be coated and made just as inefficient as the diseased parts. Further, such a crust would very soon be swept off, both by the contents of the intestine and by the increased secretion of the ulcer itself. The astringent's only influence, then, would be that of exercising a pernicious irritation on the ulcers.

We are convinced that, by the observation of the above hints, many now suffering from this grave affection may still be preserved a long time to society. Whether it is generally possible to consider these hints together, must be decided especially for each individual case. We will do well, however, to recede no farther than is absolutely necessary.—*Clinic.*

